# Release of cell wall phenolic esters during hydrothermal pretreatment of rice husk and rice straw

**DOI:** 10.1186/s13068-018-1157-1

**Published:** 2018-06-11

**Authors:** Jia Wu, Samuel R. A. Collins, Adam Elliston, Nikolaus Wellner, Jo Dicks, Ian N. Roberts, Keith W. Waldron

**Affiliations:** 1 The Biorefinery Centre, Quadram Institute Bioscience, Norwich Research Park, Colney, Norwich, NR4 7UA UK; 2 Quadram Institute Bioscience, Norwich Research Park, Colney, Norwich, NR4 7UA UK; 3 The National Collection of Yeast Cultures, Quadram Institute Bioscience, Norwich Research Park, Colney, Norwich, NR4 7UA UK

**Keywords:** Lignocellulosic biomass, Rice husk, Rice straw, Pretreatment, Inhibitors, Bio-ethanol, Phenolic esters, Ferulic acid, Di-ferulic acid

## Abstract

**Background:**

Rice husk and rice straw represent promising sources of biomass for production of renewable fuels and chemicals. For efficient utilisation, lignocellulosic components must first be pretreated to enable efficient enzymatic saccharification and subsequent fermentation. Existing pretreatments create breakdown products such as sugar-derived furans, and lignin-derived phenolics that inhibit enzymes and fermenting organisms. Alkali pretreatments have also been shown to release significant levels of simple, free phenolics such as ferulic acid that are normally esterified to cell wall polysaccharides in the intact plant. These phenolics have recently been found to have considerable inhibitory properties. The aim of this research has been to establish the extent to which such free phenolic acids are also released during hydrothermal pretreatment of rice straw (RS) and rice husk (RH).

**Results:**

RS and RH were subjected to hydrothermal pretreatments over a wide range of severities (1.57–5.45). FTIR analysis showed that the pretreatments hydrolysed and solubilised hemicellulosic moieties, leading to an enrichment of lignin and crystalline cellulose in the insoluble residue. The residues also lost the capacity for UV autofluorescence at pH 7 or pH 10, indicating the breakdown or release of cell wall phenolics. Saponification of raw RS and RH enabled identification and quantification of substantial levels of simple phenolics including ferulic acid (tFA), coumaric acid (pCA) and several diferulic acids (DiFAs) including 8-*O*-4′-DiFA, 8,5′-DiFA and 5,5′-DiFA. RH had higher levels of pCA and lower levels of tFA and DiFAs compared with RS. Assessment of the pretreatment liquors revealed that pretreatment-liberated phenolics present were not free but remained as phenolic esters (at mM concentrations) that could be readily freed by saponification. Many were lost, presumably through degradation, at the higher severities.

**Conclusion:**

Differences in lignin, tFA, DiFAs and pCA between RS and RH reflect differences in cell wall physiology, and probably contribute to the higher recalcitrance of RH compared with RS. Hydrothermal pretreatments, unlike alkali pretreatments, release cinnamic acid components as esters. The potential for pretreatment-liberated phenolic esters to be inhibitory to fermenting microorganisms is not known. However, the present study shows that they are found at concentrations that could be significantly inhibitory if released as free forms by enzyme activity.

## Background

Efficient enzymatic saccharification of lignocellulose requires pretreatment to enhance the accessibility of cellulose by cellulases [1, 2]. Pretreatments can break down cell wall bonds and loosen the cell wall polymer network [3]. Many of these processes result in the production and solubilisation of inhibitors to enzymolysis and fermentation [4, 5]. High temperatures create furan-containing moieties such as hydroxy-methyl furfural (5HMF) and furfural (2FA) from the carbohydrate components [6]. Acid, alkali and high temperatures also result in the release of many phenolics, including those derived from lignin, which can inhibit cellulases and xylanases [7–9], and cinnamic acid esters, released from hemicelluloses such as arabinoxylans that have antimicrobial activity [10]. Whilst much research has been carried out on furans and lignin-derived phenolics, the important roles of such cinnamic acid derivatives as microbial inhibitors have been highlighted only recently. Several studies have explored the potential to enhance the capability of fermenting microorganisms such as yeasts and bacteria to metabolise such phenolics [11, 12]. Sato and colleagues [13] have developed *Saccharomyces cerevisiae* using a forced evolutionary approach to increase tolerance to pCA and tFA inhibitors.

Rice straw (RS) and rice husk (RH) are globally important lignocellulosic feedstocks, particularly in China and Asian countries. Alkali pretreatment has been shown to release tFA at levels that are deleterious to microbial activity [10]. However, there has been no systematic study of the release of tFA and related moieties during hydrothermal pretreatment of RS and RH. Nevertheless, Merali et al. [1, 14] demonstrated that hydrothermal pretreatment of wheat straw resulted in considerable degradation and solubilisation of arabinoxylans and decreases in the levels of cell wall-bound phenolic esters, including ferulates and diferulates, accompanied by loss of alkaline UV turquoise fluorescence of the cell wall residues. Therefore, it is highly likely that simple phenolics may be released from rice straw and husk by hydrothermal pretreatments.

The aim of the present study has been to employ analytical HPLC with diode array detection (DAD) [14, 15] to investigate the effect of hydrothermal pretreatment severity on the release of phenolic esters, diferulates, and related moieties from RH and RS, and to establish their levels in the pretreatment liquors.

## Results

### Fourier transform infrared (FTIR-ATR) spectroscopy of untreated and hydrothermally pretreated RH and RS solids

**Fig. 1:**
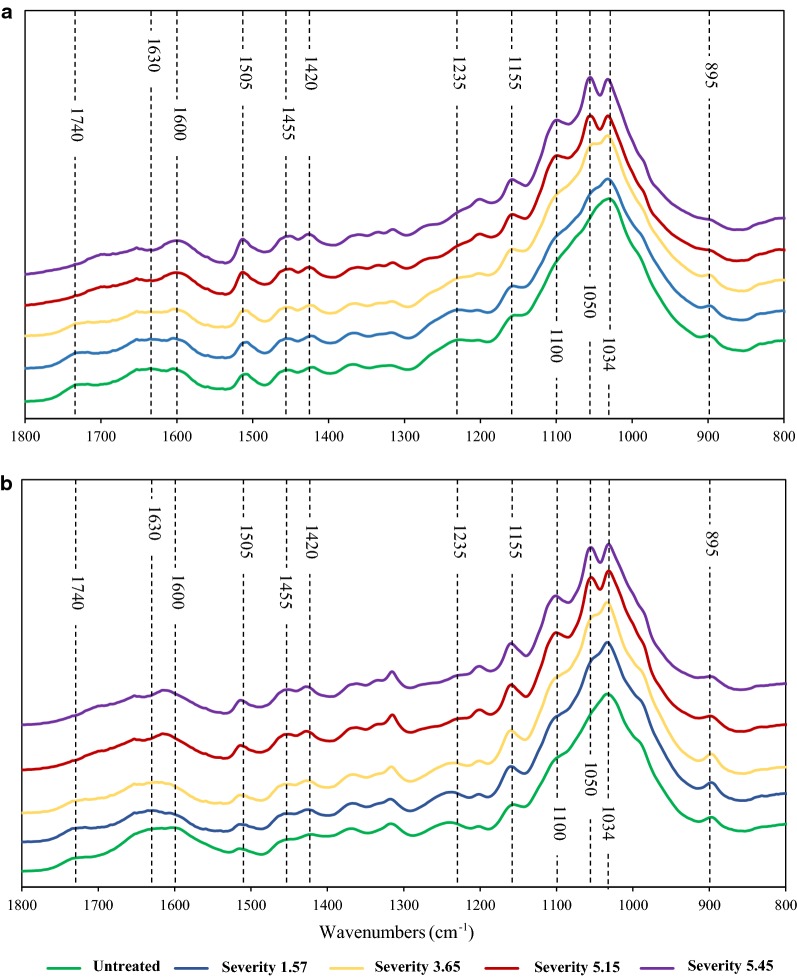
FTIR spectra of RH and RS and of their insoluble residues after hydrothermal pretreatments at different severities. **a** RH; **b** RS. Colour codes for the spectra are given below the figures

RS and RH were hydrothermally pretreated over a range of severities as described in the Methods. The pretreatment liquors were separated from the insoluble residues by centrifugation prior to analysis. General pretreatment-induced changes in cell wall composition were evaluated using the FTIR-ATR spectra region from 1800 to 800 cm^−1^. Figure 1a, b show spectra of RH and RS samples respectively; wavenumbers where pretreatment caused notable spectral changes are highlighted with vertical dashed lines. For both RH and RS, peaks at around 1740, 1630 and 1235 cm^−1^ decreased in intensity with increasing pretreatment severity. These C=O stretching and O–H bending bands relate to ester and acetyl groups of hemicellulosic polysaccharides which will have been hydrolysed and released from the residues, as shown for oilseed rape straw [16], wheat straw [3, 14, 17] and steam exploded RS and RH [18]. Associated with these losses was the increasing sharpness of carbohydrate peaks at wavenumbers around 1034, 1050, 1100 and 1155 cm^−1^ corresponding to the C–O/C–H bond stretching in cellulose, and C–O–C stretching of β-(1-4) linkages [19]. The FTIR spectra of the residues show a clear change from whole plant cell wall material towards increasingly pure lignocellulose and demonstrate the progressive removal of hemicelluloses. Lignin is mainly associated with peaks between 1600–1300 cm^−1^ and with increasing pretreatment severity, peaks became more pronounced at 1420, 1505 and 1600–1620 cm^−1^. These bands are probably due to the stretching of aromatic lignin bonds, particularly C=O and C=C bonds [19]. These results are consistent with a relative increase in the amount of lignin present in the residue after pretreatment (Table 1a).

**Table 1:** Klason lignin content (mg/g raw materials) in RH and RS samples (UT and PT); n = 3

Severity	Rice husk		Rice straw	
a Lignin content (mg/g loaded materials)				
0.00	35.25	± 1.23	22.01	± 1.37
1.57	36.18	± 1.83	24.08	± 0.82
3.65	38.89	± 1.90	26.48	± 2.38
5.15	45.57	± 1.46	34.86	± 2.92
5.45	46.22	± 0.85	36.73	± 2.35
b Lignin content (mg/g raw materials)				
0.00	35.25	± 1.23	22.10	± 1.47
1.57	34.89	± 1.77	21.95	± 0.76
3.65	31.44	± 1.59	22.17	± 2.05
5.15	34.36	± 1.17	24.89	± 2.63
5.45	32.80	± 0.67	23.61	± 0.69

a Shows lignin contents of the actual loaded biomass materials

b Shows lignin contents calculated on the basis of the original raw materials

### Fluorescence microscopy of RS and RH residues

**Fig. 2:**
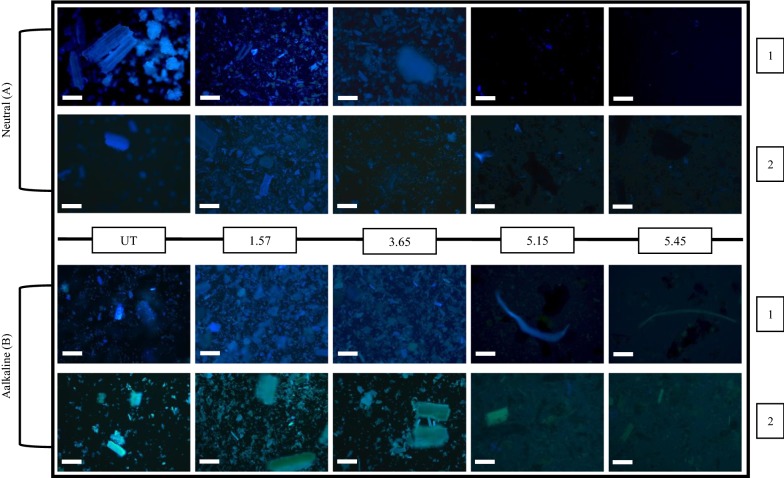
UV autofluorescence of both neutral (**a**) and alkaline (**b**) RH and RS samples (untreated UT and pretreated PT). The numbers 1 and 2 represent RH samples and RS samples respectively. In **a** (neutral), only blue autofluorescence occurs, symptomatic of lignin and pCA. In **b** (alkali), the RH autofluoresces blue, whilst the RS autofluorescencence is turquoise/green symptomatic of tFA and associated moieties. Scale bar: 100 µm

The visual appearance of lignin and phenolic acids in the RH and RS (untreated and pretreated) was obtained using UV autofluorescence under neutral (Fig. 2a) and alkaline conditions (Fig. 2b). As Fig. 2a (1 and 2) shows, lignin and phenolic acids were all in blue under neutral condition and the levels of fluorescence were not significantly different between RH and RS samples. Under alkaline condition, RH was predominantly blue in colour (symptomatic of lignin) (Fig. 2b (1)) whilst RS was green/turquoise reflecting significant levels of cinnamic acid derivatives such as ferulic acid (tFA) [20] and relatively lower levels of lignin (Fig. 2b (2)) (see below). As pretreatment severity increased, the loss of fluorescence was observed under both neutral and alkaline condition (Fig. 2), suggesting the removal of lignin and phenolic acids after pretreatment.

### Quantification of Klason lignin in the solids of nontreated and pretreated RH and RS

The content of Klason lignin in RH and RS was measured (Table 1). RH contains considerably more lignin than RS in untreated and pretreated samples. Table 1a shows increases of lignin content in residues of both RH and RS after pretreatment especially at higher severities. This is consistent with the hydrothermal release of hemicellulosic polysaccharides and volatile chemicals such as furfural as found in many other pretreatment studies [21, 22]. The concomitant increase in lignin is inconsistent with the pretreatment-related decline in fluorescence of the residues shown in Fig. 2 and suggests that fluorescent moieties, probably at the surface of the lignified material, had been lost disproportionately. Table 1b shows that the lignin remaining in the residues, when presented as a function of the original raw material, is not significantly altered after hydrothermal pretreatments.

### Investigation of phenolic compounds in the liquors of pretreated samples

**Fig. 3:**
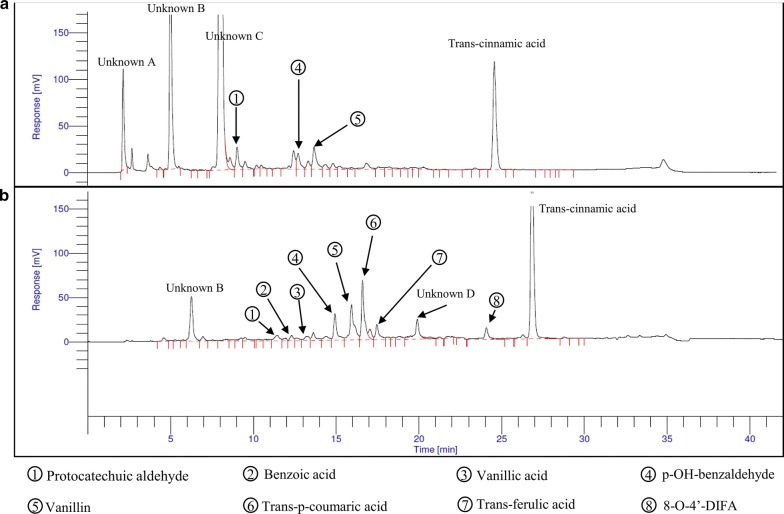
HPLC chromatogram of phenolic compounds in RS pretreatment liquor produced at a severity of 5.15. **a** Direct injection of liquor showing the presence of only pCald, *p*-OH-Bzald and vanillin; **b** HPLC of moieties recovered by liquid–liquid extraction after saponification (showing a wide range of identified phenolics)

Initial attempts to quantify tFA, diferulic acids (DiFA) and related phenolics that may have been released by hydrothermal pretreatment (severity 5.15), involved direct analysis of the pretreatment liquor by HPLC–DAD (Fig. 3a). Several large, unidentified early-running peaks (A, B and C) were detected. However, the only free phenolics that could be identified from pure standards were protocatechuic aldehyde (pCald), *p*-OH-benzaldehyde (*p*-OH-Bzald) and vanillin. To assess the presence of esterified phenolics, the pretreatment liquor was subjected to saponification (1 M NaOH) followed by liquid–liquid extraction and HPLC [15]. The results (e.g. Fig. 3b) revealed a wide range of phenolics that could be separated by HPLC and identified from their retention times relative to the *trans*-cinnamic acid internal standard, and diode-array recorded spectra. Interestingly, saponification reduced the levels of early-running unidentified moieties in Fig. 3a (Unknown A, B and C peaks). Thus, it appears that the phenolics present in the pretreatment liquor were probably esterified to rapidly eluting fragments of polysaccharides that had been released by pretreatment-induced autohydrolysis. Hence, the saponification method was chosen for identification and comparative analysis of phenolic acids in the pretreatment liquors. After the investigation of liquors of pretreated samples, the phenolics remaining esterified to the hydrolysate solids were also extracted and assessed using the same approach [15].

### Comparison of total phenolics extracted from solids of RH and RS (untreated and pretreated) after saponification

For extracting the phenolics from pretreated hydrolysate solids, preliminary studies showed that 4 Mol NaOH for 17 h was more effective for extracting phenolic esters than 1 Mol NaOH (Table 2) from raw RH and RS residues. However, for pretreated residues, 1 Mol was as effective. Therefore, to avoid unnecessary alkaline degradation, phenolics from the pretreated liquors and residues were saponified using 1 Mol NaOH for 17 h prior to acidification and liquid–liquid extraction.

**Table 2:** Quantification of total phenolic compounds extracted from the solids of untreated and pretreated (severity 1.57) RH and RS

Severity	Total (mono) phenolics (mg/g of raw materials)							
	Rice husk				Rice straw			
	1 Mol NaOH		4 Mol NaOH		1 Mol NaOH		4 Mol NaOH	
Raw	14.57	± 0.57	15.82	± 1.41	14.37	± 0.45	17.20	± 1.57
1.57	15.90	± 0.44	15.96	± 1.21	16.23	± 0.78	14.24	± 0.83

Untreated and pretreated samples were saponified with 1 Mol NaOH and 4 Mol NaOH separately before analysis by HPLC

Results were calculated as mg/g of original lignocellulosic raw materials. n = 3

### Phenolic compounds in the solids and liquors of raw and pretreated RH and RS

**Fig. 4:**
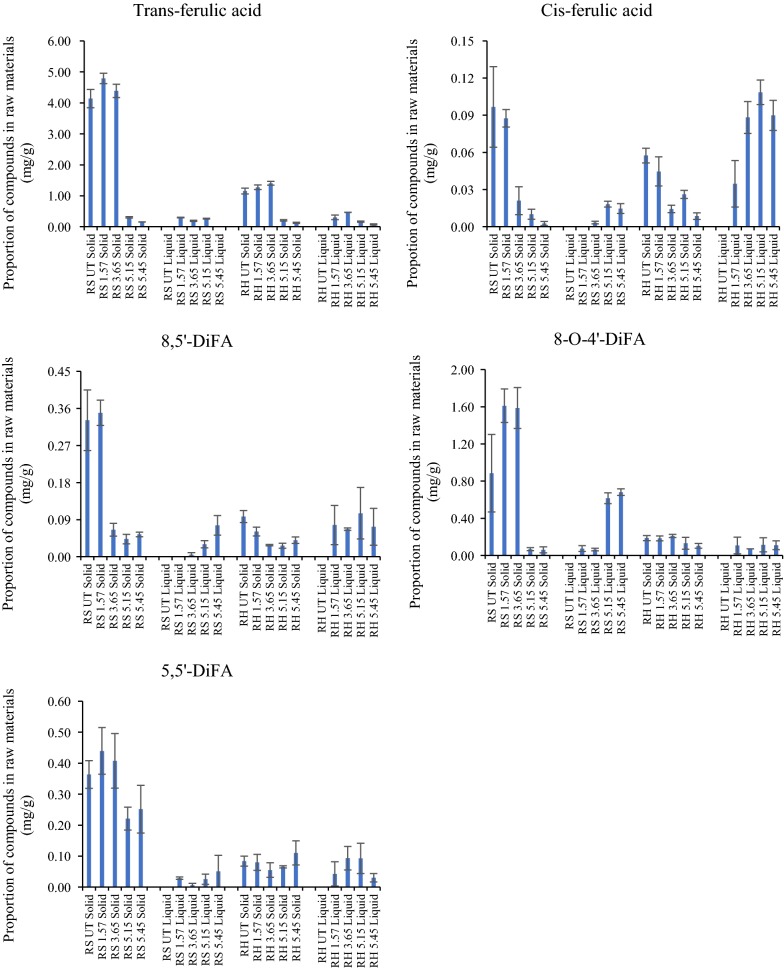
Ferulic acid and diferulic acids quantified after saponification of the solids and liquors of pretreated RH and RS. Yields were calculated as mg/g dry matter of initial raw materials; n = 3

**Fig. 5:**
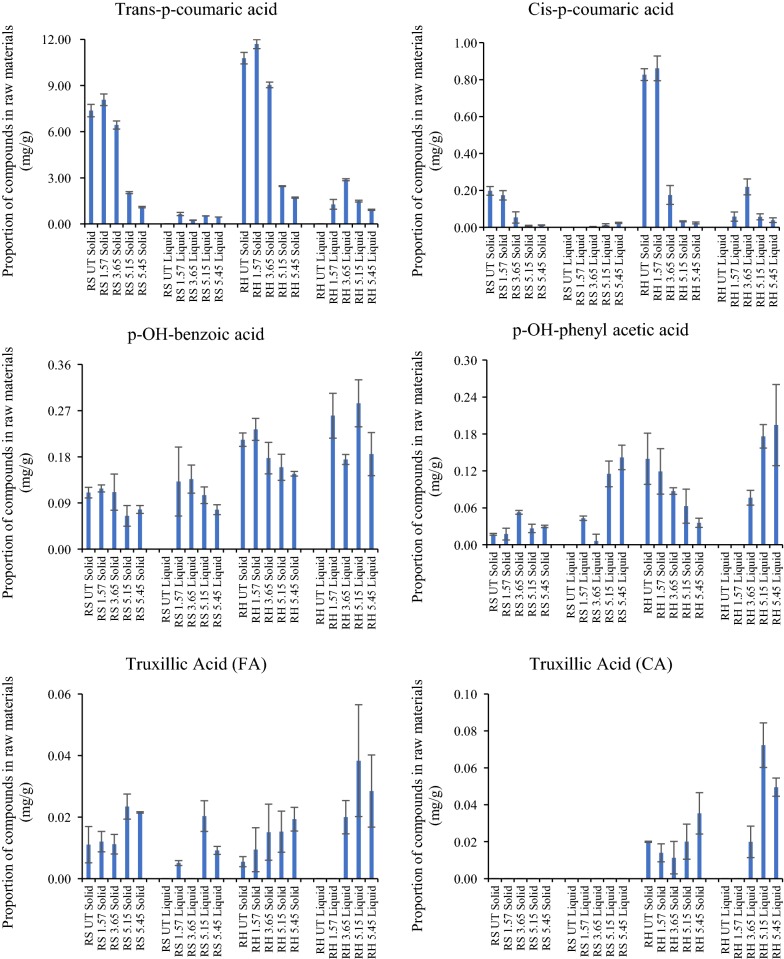
Phenolic acids quantified after saponification of the solids and liquors of pretreated RH and RS. Yields were calculated as mg/g of dry matter of initial raw materials; n = 3

**Fig. 6:**
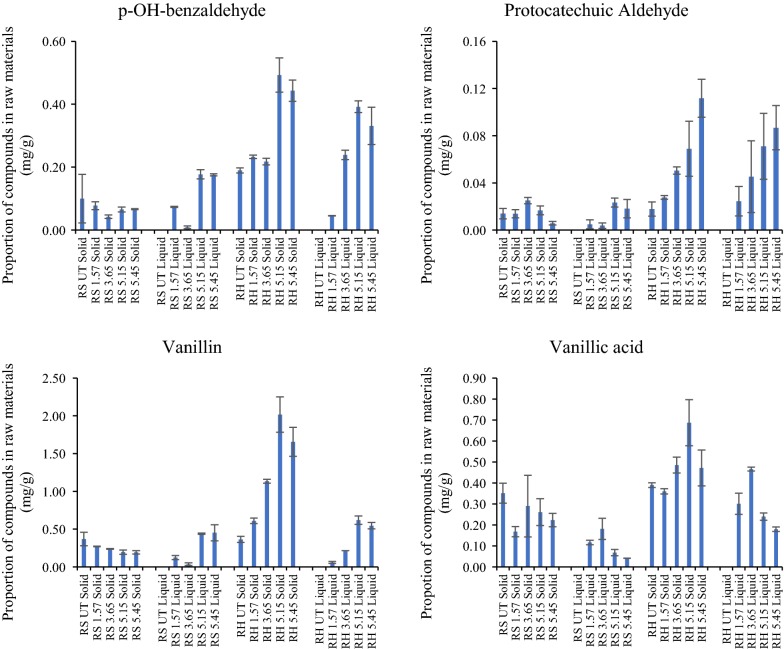
Phenolic compounds quantified after saponification of the solids and liquors of pretreated RH and RS. Yields were calculated as mg/g of dry matter if initial raw materials; n = 3

In pretreated liquors and residues of RH and RS, 15 different phenolic compounds were identified and quantified including 12 phenolic acids, 2 aldehydes and 1 vanillin (Figs. 4, 5, 6). Total yields calculated for phenolics recovered from insoluble residues and the separated liquors after pretreatment at different severities are shown in Table 3. Total phenolics were similar in untreated RH and RS and in PTRH and PTRS pretreated at severity of 1.57. When severity was increased, the contents of phenolic compounds remaining in RH and RS residues were significantly reduced, and RH contained more total phenolics than RS in samples pretreated at severities of 3.65, 5.15 and 5.45.

**Table 3:** Contents of total phenolic compounds in the solids and liquors of untreated and pretreated RH and RS

Severity	Phenolic compounds (mg/g raw materials)					
	RH			RS		
	Solids	Liquors	Total	Solids	Liquors	Total
ut (4 M)	15.82 (± 1.27)	N/A	15.82	17.20 (± 1.57)	N/A	17.20
1.57	15.90 (± 0.44)	2.59 (± 0.60)	18.43	16.23 (± 0.78)	1.56 (± 0.20)	17.93
3.65	13.13 (± 0.28)	5.14 (± 0.15)	18.28	13.73 (± 0.65)	0.91 (± 0.05)	14.93
5.15	6.50 (± 0.50)	4.02 (± 0.11)	10.52	3.37 (± 0.21)	2.46 (± 0.44)	5.83
5.45	5.07 (± 0.43)	2.94 (± 0.24)	8.01	2.27 (± 0.20)	2.22 (± 0.09)	4.49

Results were calculated as mg/g of raw materials. n = 3

Identifiable ferulic acid moieties are presented in Fig. 4. tFA was present in considerably higher quantities in RS compared with RH. Pretreatment of both substrates resulted in little change in yields at the lower severities. However, at the higher severities, the levels of extractable tFA decreased by over 85%. Interestingly, whilst a small quantity of (esterified) ferulic acid could be detected in the liquors, this was at relatively low levels. Small levels of *cis*-FA were detected, and these showed a decrease in the residues at higher pretreatments, but an increase in the liquors. Three diferulic acid moieties were also identified in untreated and pretreated RS and RH for the first time, released by saponification from both residues and liquors. The most abundant was 8-*O*-4′-DiFA, followed by 5,5′-DiFA and then 8,5′-DiFA. Generally, the DiFAs showed similar trends to tFA, in that larger quantities were present in the RS residues than those of RH and decreased from the residues at the higher pretreatment severities. Under these conditions, they increased in the pretreatment liquors and, unlike tFA, were maximum in the liquors obtained at higher severities suggesting a much higher degree of thermal stability.

In contrast to the ferulates above, all other simple phenolics extracted and quantified were, except for tFA-derived truxillic acid (Fig. 5), present at higher levels in the RH and its liquors compared with RS. The most prominent of these was *para*-coumaric acid (pCA; Fig. 5) which was present at much higher levels than tFA generally. The levels of both pCA and tFA decreased in the residues as pretreatment severity increased. The other phenolics comprised truxillates (tFA- and pCA-derived), *p*-OH-benzoic acid, *p*-OH-phenyl acetic acid (Fig. 5), *p*-OH-benzaldehyde, protocatechuic aldehyde, vanillin and vanillic acid (Fig. 6). Interestingly, the levels of *p*-OH-B, truxillic acid (FA-derived), PA, vanillin, *p*-OH-Bzald and pCald increased in both the pretreated RH and RS residues at the higher severities. It is possible that they are hydrothermally derived breakdown products from other wall phenolics, for example, those in lignin (hence the higher levels in RH). Recently, Rasmussen et al. [23] have shown that hydrothermal pretreatments can create a range of oligophenolic enzyme inhibitors from wheat straw lignocellulose.

## Discussion

Lignin, lignin-derived phenolic compounds, hemicellulose and cellulosic saccharide breakdown products significantly reduce the efficiency of production of cellulosic bio-ethanol [24–26]. Several phenolic compounds have been reported to be released from lignocellulosic biomass during pretreatment including phenolic acids, tannins and gallic acid [27–29]. Other substantial research has highlighted the importance of lignins and lignin-derived phenolics in cell wall interpolymeric crosslinking (e.g. Sun et al. 2001). This study has extensively investigated the release and degradation of simple (esterified) cell wall phenolic compounds during hydrothermal pretreatment across a range of severities and has provided new information on the fate of diferulic acids.

Only recently has the inhibitory role of phenolic esters such as tFA and *p*-CA been considered seriously. Much of that work has focused on the free phenolic acids released after alkali pretreatments [10]. Such studies have demonstrated that ethanol producing strains of *E. coli* exhibit IC_50_ values of about 2.5 mM each for free tFA and pCA. In the present study, no free tFA, pCA or diferulate phenolic acids were detected in the pretreatment liquors (Fig. 3). However, if the esterified phenolics present in the RH pretreatment liquors were to be de-esterified by esterases in the cellulase cocktails or by esterases released from the fermenting organisms, the resulting free tFA and pCA could reach concentrations of 0.12 and 0.9 mM, respectively—levels that would be significantly inhibitory to microbial fermenting organisms [10]. Currently, there is no information on their inhibitory functionality in the soluble, esterified forms, and further work will be needed to establish this. Also, there is no information currently on the potential inhibitory activity of the solubilised diferulate esters. Free diferulates may also be of significance in alkali pretreatment liquors.

After the lower severity pretreatments (which are in the commercial user range), significant levels of phenolic esters remain attached to cell wall polymers (Fig. 2). Whilst such moieties are unlikely to directly affect microbial activity, they may additionally function in inhibiting alcohol production. For example, the DiFAs create interpolymeric cross-links between arabinoxylan hemicelluloses. Phenolic esters are also known to cross-link polysaccharides with lignin [30, 31]. Their presence is likely to attenuate hemicellulose disassembly and solubility during pretreatment and reduce subsequent diffusion of cellulases and hemicellulases into the wall matrix. Furthermore, some DiFAs have been strongly implicated in cell adhesion [20, 32, 33] and may influence the rate and extent of cell separation in cereal residues during hydrothermal pretreatments as indicated previously [14]. This latter property is likely to affect the pretreatment-induced increase in surface area-to-volume ratio of pretreated particulates. The levels of simple phenolics in RS and RH are also likely to have implications in relation to digestibility by ruminants. pCA had been reported to be associated with inhibitory activities reducing the digestibility of cell wall carbohydrates [34], and has been implicated as a toxin to microorganisms and a barrier to the digestion of materials during simulated rumen fermentation [35].

Finally, the majority of total phenolics from both RH and RS samples were degraded and lost after pretreatment at the higher severities. Such degradation of potential inhibitors may have a positive impact on saccharification and fermentation.

## Conclusion

Hydrothermal pretreatment of RH and RS resulted in a decrease in hemicelluloses and a concomitant increase in the levels of cellulose and lignin. Simple phenolics such as tFA, diferulates and pCA were present in RH and RS; and were released, probably as esters of cell wall polysaccharide fragments, into the liquor during pretreatment, and degraded at the higher severities. Differences in lignin, tFA, DiFAs and pCA between RS and RH reflect differences in cell wall physiology and are probably responsible, in part, for the higher recalcitrance of RH. The potential for pretreatment-liberated esterified phenolics to be inhibitory to fermenting microorganisms is not known. However, they are at concentrations that could be significantly inhibitory if released by enzyme activity. In addition, the release of other free phenolics such as vanillin, *p*-Cald, *p*-OH-B and *p*-OH-Bzald during pretreatment may also reduce the efficiency of saccharification and fermentation.

## Materials and methodology

### Materials

RS and RH of the same variety were sourced as described previously by Wood et al. [18].

### Milling of rice husk and rice straw

RH and RS (prechopped into about 2 cm lengths) were milled into small particles (< 0.5 mm) by using RETSCH cyclone mill (Retsch Limited, Hope Valley, United Kingdom). Milled materials were collected into plastic sample pots sealed with screw-caps and then stored under lab condition.

### Thermodynamic pretreatment of rice husk and rice straw

Pretreatments of milled RH and RS were carried out using a BIOTAGE® Initiator and Reactor (Biotage AB, Uppsala, Sweden). Pretreatment severity was introduced and adapted from the research of Overend et al. [36].

$${\text{Severity}}\,({\text{Ro}}) = \log_{10} (t.{ \exp }^{{\frac{T - 100}{14.75}}} )$$

Severity was calculated from temperature and duration. Four different severities: 1.57 (140 °C, 2.5 min), 3.65 (190 °C, 10 min), 5.15 (200 °C, 160 min), 5.45 (210 °C, 160 min) were selected to pretreat milled RH and RS [37]. To give a 5% (w/w) suspension, 750 mg of each sample was transferred into 20 ml microwave pressure tubes respectively and followed by the addition of 14.25 ml distilled water. Those tubes were then capped and pretreated by using the Biotage reactor. After the pretreatment process, those tubes were cooled with compressed air to ambient and then stored in freezer (− 20 °C).

### Fluorescence microscopy of pretreated and untreated RH and RS slurries

Pretreated slurries (containing both liquids and solids) were defrosted and centrifuged. The supernatants were removed from pellets and transferred to 15-ml plastic tubes for further investigation. For the neutral set, sample residues were re-suspended into distilled water, and for the alkaline set, samples of each residue were then treated with 1% NaOH (w/v) to establish an alkaline environment, and then autofluorescence was assessed immediately using an Olympus BX 60 fluorescence light microscope (Olympus, Tokyo, Japan) equipped with a Progress C10^plus^ camera and software. Autofluorescence of each sample was recorded three times using a UV filter cube U-MWU, exciter filter BP330-385, and barrier filter BA 420.

### Fourier transform infrared (FT-IR) of pretreated and untreated RH and RS solids

Pretreated solids of RH and RS were separated from liquors and oven-dried at 65 °C overnight. FT-IR spectra of each sample were collected using a BioRad FTS 175C Fourier transform infrared spectrometer (BioRad, Cambridge, MA, USA). Milled raw RH and RS and dried solids of pretreated RH and RS were placed in a Golden Gate™ diamond-attenuated total reflectance (ATR) accessory (Specac, Slough, UK). Triplicates of each sample were scanned 100 times at a resolution of 2 cm^−1^ and the spectra were averaged and referenced against a spectrum of the empty crystal. The spectra were collected in the region of 4000–800 cm^−1^, were truncated to 1800–800 cm^−1^ and area normalised for analysis.

### Klason lignin analysis of pretreated rice husk and rice straw

Pretreated slurries (containing both liquids and solids) of RH and RS were oven-dried at 65 °C overnight, and then 100 mg of each sample was loaded into 25-ml Sovirel culture tubes (The Science Company, 7625 W Hampden Ave, Unit 14, Lakewood, Colorado, US). The hydrolysis procedure was started at room temperature with the additions of 1.5 ml sulphuric acid. After 3-h incubation, 18 ml distilled water was added to each tube, and they were incubated at 100 °C for 2.5 h. Hydrolysates of RH and RS were then transferred into pre-weighed sintered glass funnels (WT funnels) with porosity four (VWR International Ltd, 1151 Budapest, Szövőgyár utca 11–13, Hungary) and then washed with distilled water until the acid was completely removed. The funnels containing the residues were dried at 50 °C overnight and weighed, then placed into a Vulcan PD Furnace 3–550 (Dentsply Sirona Global Headquarters, Susquehanna Commerce Center. 221 West Philadephia Street, Suite 60 W, York PA, USA) and incinerated at 500 °C for 22 h. The weights of funnels containing ash were recorded (WT funnels and ash). Samples for lignin analysis were prepared as triplicates. Final lignin contents of samples were calculated using the following equation:

$${\text{Lignin }} = {\text{ WT funnels and hydrolysates }}{-}{\text{ WT funnels and ash }}\left( {{\text{mg}}/{\text{g Raw materials}}} \right).$$

### Analysis of phenolic compounds in untreated and pretreated RH and RS solids

Liquors of pretreated RH and RS were transferred into tubes and stored at − 20 °C for analysis. Solids were dried at 65 °C overnight and 5 mg of each sample was loaded into Sovirel tubes. Saponification was carried out by addition of 4 ml 1 M NaOH (de-oxygenated with nitrogen). After de-oxygenating by over-flushing nitrogen, the tubes were capped with screw caps and placed in the dark on a rotating sample mixer for 21 h. At the end of this period the samples were neutralised and acidified by adding 1.5 ml distilled water and 0.5 ml of concentrated HCL (37% w/v). *Trans*-cinnamic acid (0.2 mg/ml, dissolved in 1:1 Methanol–water mixture) was used as internal standard and 50 µl was added into each sample. Liquid–liquid extraction of phenolic acids from the acidified solution was carried out by using ethyl-acetate (three times). Following the evaporation of ethyl-acetate, phenolic acids were re-dissolved in 1 ml methanol–water mixture. Phenolic acids were analysed and quantified by using HPLC (High-Performance Liquid Chromatography) using a Perkin-Elmer series 200 LC Pump, Perkin-Elmer advanced LC Processor ISS200, Phenomenex Column Luna 5 µ C18 (2), 250 * 4.6 mm equipped with precolumn and Perkin Elmer Diode Array UV Detector (Waltham, Massachusetts, USA) [15]. Phenolic compounds were initially identified by their relative retention time and then further compared to identical chromatography spectrum of individual phenolic compounds. The method for identifying phenolics was adapted from the study of Waldron [38].

Solids of untreated and samples pretreated at severity 1.57 were saponified as above but using 4 M NaOH. Samples were analysed in triplicate.

### Phenolic compounds analysis of liquors of pretreated RH and RS

Method A (direct injection): 50 µl of 0.2 mg/ml internal standard (*trans*-cinnamic acid) was added to a sample tube containing 95 µl of liquor and then 855 µl of methanol (50% v/v) was added to give a total volume of 1 ml. This was injected directly onto the HPLC–DAD.

Method B: Saponification and liquid–liquid extraction. This followed the method for extracting and analysing esterified phenolic acids of pretreated solids (above). The same method and HPLC was used for the quantification of phenolic acids.

Samples were all prepared and processed as triplicates.

## Abbreviations

RH: rice husk
RS: rice straw
PTRH: pretreated rice husk
PTRS: pretreated rice straw
HPLC: high-performance liquid chromatography
tFA: *trans*-ferulic acid
pCA: *para*-coumaric acid
DiFA: diferulic acid
5HMF: hydroxymethalfurfural
2FA: furfural
pCald: protocatechuic aldehyde
VA: vanillic acid
*p*-OH-B: *p*-OH-benzoic acid
DAD: diode array detection
FTIR-ATR: fourier transform infrared–attenuated total reflectance
